# Variability in Diagnosis and Treatment of Kawasaki Disease across the United States

**DOI:** 10.1016/j.jpedcp.2026.200237

**Published:** 2026-08-17

**Authors:** Chisato Shimizu, Laurel DeHaan, Shoko Yamazaki, Kyohka Sato, Daniel R. Cayan, Jennifer Kim, Emelia V. Bainto, Kirsten B. Dummer, Adriana H. Tremoulet, Jane C. Burns, Michael A. Gardiner

**Affiliations:** 1 Department of Pediatrics, School of Medicine, University California San Diego, San Diego, CA; 2 Rady Children's Hospital San Diego, San Diego, CA; 3 Scripps Institution of Oceanography, University of California San Diego, San Diego, CA

**Keywords:** coronary artery aneurysms, seasonality, adjunctive therapy

## Abstract

**Objective:**

To analyze demographic characteristics, treatment, and outcome of patients with Kawasaki disease (KD), an acute vasculitis of children with unknown etiology, to gain insight into variation in care and epidemiologic clues regarding the etiology of KD.

**Study design:**

We analyzed the first admission of children diagnosed with KD and treated with intravenous immunoglobulin (IVIG) between 2015 and 2019 across the US and studied the monthly diagnosis of KD by age group and geographic location. We evaluated the use of adjunctive therapies, including patients with giant coronary artery aneurysms (gCAAs) who were treated with systemic anticoagulation after IVIG treatment.

**Results:**

We identified 8037 patients with acute KD in 45 hospitals. The seasonal cycle of patients ≤6 months old differed from that of other age groups, with regional variability noted between the Western and Southern US. The rate of adjunctive therapy use ranged from 14.8% to 85.6% at individual hospitals. There was significant variability in the choice of adjunctive agents, with regional differences in the use of corticosteroids and infliximab. There were 138 patients with gCAAs (1.7%), of whom 42 (30.4%) received no additional anti-inflammatory therapies beyond IVIG, despite 26.8% of these aneurysms being present at the time of diagnosis.

**Conclusions:**

Distinct temporal signatures of KD diagnosis across age groups suggest different age-related mechanisms of exposure. Significant practice variation exists in the use of adjunctive therapy in patients with KD with and without gCAAs. Future head-to-head comparisons of adjunctive agents in high-risk patients will be necessary to guide best practice.

Kawasaki disease (KD) is an acute systemic vasculitis in children and the leading cause of pediatric acquired heart disease. Currently, there is no active U.S. surveillance program for patients with KD. Therefore, administrative databases are the only source to examine trends in KD incidence and treatment over time.^1^, ^2^, ^3^ Using a large Japanese cohort (>420 000 patients), our group previously demonstrated distinct age-specific patterns of KD incidence that suggested exposures to different KD triggers that varied by age group.^4^ No large-scale analysis of the age-specific seasonal incidence of KD has been reported in the US. Such an analysis could lead to new insights into KD epidemiology and provide clues to the etiology.

The gold standard initial therapy for acute KD is intravenous immunoglobulin (IVIG) with aspirin. Treatment with IVIG reduces the incidence of coronary artery aneurysms (CAAs) from ∼25% to less than 5%.^1^ Despite appropriate initial treatment, 10%-20% of patients with KD develop a recrudescent or persistent fever after completion of their IVIG infusion (IVIG-resistance). Despite initial IVIG therapy, some patients develop giant aneurysms (gCAAs) defined by a *z* score (coronary artery internal diameter normalized for body surface area) ≥10 and are at the highest risk for myocardial infarction even decades after the onset of KD.^5^, ^6^, ^7^ The recent American Heart Association (AHA) statement recommends adjunctive anti-inflammatory therapies be added to IVIG for patients who demonstrate IVIG-resistance or who are considered to be at high risk for developing CAAs (≤6 months of age or left anterior descending or right coronary artery internal diameter normalized for body surface area ≥2.5 SD units on baseline echocardiography).^8^ Currently, there are limited data from large-scale clinical trials in mixed-ethnic populations in the US to guide clinicians in the choice of therapeutic agents for children with IVIG resistance or those at high risk of developing CAAs.^9^ Accepted therapies include corticosteroids, infliximab (IFX), repeat IVIG infusion, anakinra (ANA), and cyclosporine.^8^

The Pediatric Health Information System (PHIS) collects all data associated with financial charges from nonprofit, tertiary care pediatric hospitals affiliated with the Children's Hospital Association (Lenexa, KS, https://www.childrenshospitals.org/). The participating hospitals in the PHIS are the largest and most advanced children's hospitals in the US and represent the highest standard of pediatric care. Therefore, the PHIS provides a unique snapshot of KD patient care and outcomes across the US.

In this study, we used PHIS data to gain a snapshot of the seasonal diagnosis of KD in the US by age and geographic region, examine practice variability in the adjunctive treatment of patients, and assess the treatment of children with presumed gCAAs.

## Methods

We identified inpatients treated for acute KD with an admission date from January 1, 2015, to December 31, 2019, to avoid confusion with Multisystem Inflammatory Syndrome in Children during the COVID-19 pandemic and to include the largest number of hospitals. Hospitals contributing at least 3 years of data during the 5-year study period were included. Among the 45 included hospitals, 42 had complete data, while 3 lower volume hospitals (10-20 patients per year) had 3-7 quarters of missing data during 2015-2016.

We included children of any age with a diagnosis of acute KD (*International Classification of Diseases* [ICD] 9: 446.1 and ICD-10: M30.3) who were treated for the first time during the study period with one or more doses of IVIG (Figure 1). We collected data including age, sex, ethnicity, hospital of admission, intensive care unit admission, number of echocardiograms, need for mechanical ventilation, and dates of admission and discharge. We recorded hospital treatments including second IVIG, corticosteroids, IFX, etanercept, methotrexate, ANA, cyclosporine, and cyclophosphamide. We also recorded the use of anticoagulants, including warfarin, enoxaparin, and apixaban. To avoid confusion with an initial IVIG infusion spanning multiple dates, or initial treatments that were paused and restarted, we defined repeat IVIG treatment as a second IVIG infusion billed at least 2 days after the first infusion. A readmission within 7 days of the initial discharge was considered to be related to the same KD episode.

**Figure 1:**
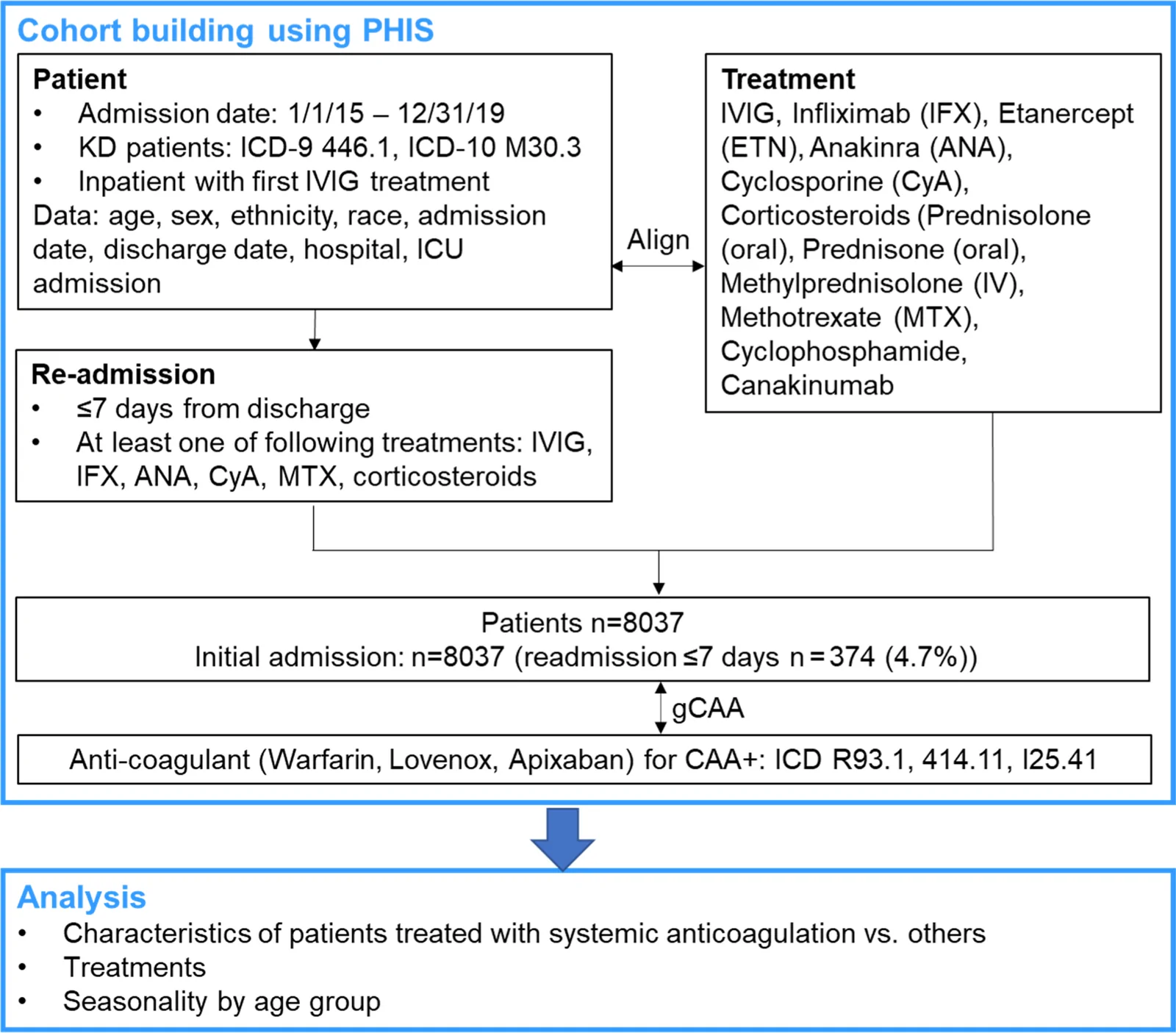
Study workflow.

### Seasonality of KD by Age Group and Region

Using the dates of admission, we computed the seasonality of KD based on a time series smoothed with a 31-day running mean by finding the percentage of patients with KD admitted on each date and smoothing that count with a 31-day running mean (the mean was found for the value of each day with the 15 previous days and 15 subsequent days). We used a 31-day running mean to reduce the effect of the day-to-day variation in the record. An even distribution of cases would result in 0.27% of cases each day (100% of patients with KD/365 days per year). Unique seasonal cycles were computed for 4 age groups: ≤6 months, >6 months and ≤2 years, >2 and ≤3 years, and >3 years. Similarly, unique seasonal cycles were also computed for 4 census regions. These regions were established to prevent the identification of individual hospitals by aggregating the data.

### Diagnosis of CAAs

Patients coded as having “abnormal findings on diagnostic imaging of heart and coronary circulation (ICD R93.1),” “aneurysms of coronary vessels (ICD-9 414.11),” or “coronary artery aneurysms (ICD-10 I25.41)” were considered to have CAAs. Based on AHA-recommended KD management, patients with CAAs who were treated with any anticoagulant after IVIG treatment were considered to have gCAAs (*z* score ≥10 for the right or left anterior descending coronary arteries). Due to anticipated inaccuracy in the diagnosis of CAAs based on ICD-9/ICD-10 codes alone, we focused our analysis on those with CAAs with anticoagulation who were more accurately identifiable from the available PHIS data using treatment with systemic anticoagulation after IVIG treatment as a proxy for gCAAs.

### PHIS Data Validation

To perform a quality check on the coding of patients with KD in the PHIS database, we compared PHIS data from our center with our existing KD Research Electronic Data Capture (REDCap) database hosted by UCSD. This REDCap database is curated by a dedicated database manager and reviewed by the clinical team to ensure accuracy. We evaluated the number of patients who were incorrectly classified by PHIS, missing from the PHIS query, and identified as developing CAAs and gCAAs.

### Statistics

We used χ² tests for categorical values and 2-tailed *t* tests for continuous variables, with *P* < .05 considered significant. For seasonal cycle analyses, we used random sampling to test for significant differences between seasonal cycles. For each partial seasonal cycle (single age group or region), 1000 random seasonal cycles were created by selecting a sample of the same size as the partial group from the entire study population. The actual seasonal cycle was compared with the randomly selected samples. Days when the actual cycle was in the highest or lowest 2.5% were considered significantly different from the entire sample (*P* < .05).

## Results

### Demographic and Clinical Characteristics

We identified 8037 patients with acute KD hospitalized and treated with IVIG over the 5-year period in 45 hospitals (Figure 1) located in 26 states across all 4 census regions of the US. Demographic and clinical characteristics of patients at initial admission are shown in Table I. The median age was 2.9 years (IQR, 1.5-5.0 years), and 60% were male patients. Intensive care unit admission (9.1%) and mechanical ventilation (1.4%) were uncommon.

**Table I:** Demographic and clinical characteristics of the 8037 patients with KD hospitalized at 45 free-standing children's hospitals

Characteristics	n (%) Unless specified
Male	4814 (60)
Median age in years (IQR)	2.9 (1.5-5.0)
Age group	
≤6 mo	469 (5.9)
>6 mo and ≤24 mo	2405 (29.9)
>24 mo and ≤36 mo	1302 (16.2)
>36 mo	3861 (48.0)
Race and ethnicity	
White, non-Hispanic	2876 (36)
Hispanic	1748 (22)
Black	1580 (20)
Other, mix or unknown	1023 (12)
Asian	810 (10)
Year of onset	
2015	1589 (19.8)
2016	1585 (19.7)
2017	1651 (20.5)
2018	1644 (20.5)
2019	1568 (19.5)
Census region∗	
West	2220 (27.6)
Midwest	1712 (21.3)
South	3062 (38.1)
Northeast	1043 (13.0)
Days of hospitalization, n (IQR)	4 (3-5)
Number of echocardiograms during hospitalization, n† (%)	
1	5222 (68)
2	1603 (21)
≥3	653 (5.0)
Missing info	224 (2.9)
ICU	728 (9.1)
Mechanical ventilation	111 (1.4)
Additional anti-inflammatory treatment	
None	5582 (70)
Additional IVIG	741 (9)
Corticosteroids	662 (8)
Additional IVIG plus corticosteroids	411 (5)
Infliximab	319 (4)
Other combinations	322 (4)

∗ Census region determined by PHIS.

† Two hospitals were excluded due to missing >50% of echocardiography data.

### Seasonality of KD

We explored the seasonality of KD by age group and geographic region. Patients were divided into 4 groups: ≤6 months, >6 months and ≤2 years, >2 and ≤3 years, and >3 years (Figure 2, A). Overall, the percentage of new KD diagnoses per day (the number of new patients on a given day of the year divided by the total number of patients for each year) peaked in February and was lowest between July and September for patients >6 months (Figure 2, B). However, the seasonality for infants ≤6 months showed significant differences from the other age ranges, with a peak in August and September and a nadir in January and February (Figure 2, B).

**Figure 2:**
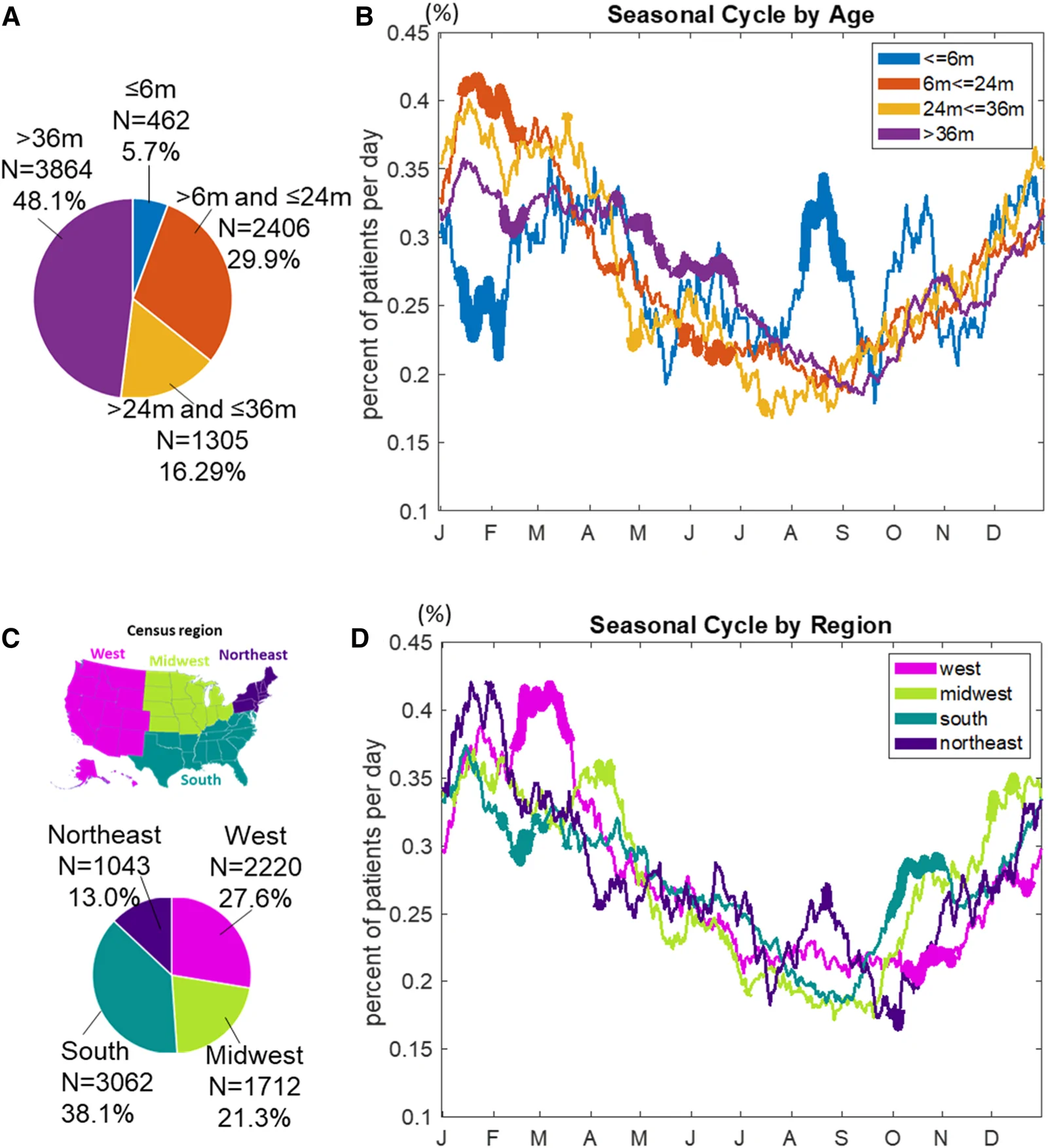
Age-specific and regional differences in the seasonality of KD incidence. **A,** Numbers and percentage of the patients in the 4 groups (≤6 months, >6 months and ≤2 years, >2 and ≤3 years, and >3 years) are shown in the pie chart. **B,** Seasonal cycles by age. A heavy line: Days when a given age group is significantly different from the others (*P* ≤ .05). **C,** Locations of the 4 census regions in the US map. Numbers and percentage of the patients in the 4 census regions are shown in the pie chart. **D,** Regional differences in seasonality.

Seasonal cycles were also computed for 4 census regions: West, Midwest, South, and Northeast (Figure 2, C). Overall, the rate of diagnosis was highest in January to March and lowest in September to October (*P* < .05) (Figure 2, D). However, there were significant differences in the seasonal cycles between the West and South census regions. In March, the KD rate increased in the West and decreased in the South. Reciprocally, in October, the KD rate increased in the South and decreased in the West (*P* < .05). These differences were not driven by age differences between the 2 regions, as patients aged ≤6 months accounted for 7.1% (19 of 268) in the South and 7.2% (10 of 138) in the West in October.

### Variation in Adjunctive Treatments

The majority of patients with KD were treated with a single infusion of IVIG [5582/8037 (70%)] (Table I). The remaining 30% of patients received additional anti-inflammatory therapies, including second IVIG infusion, corticosteroids, second IVIG plus corticosteroids, IFX, and other combinations that included ANA (n = 43), cyclosporine (n = 42), cyclophosphamide (n = 10), methotrexate (n = 9), etanercept (n = 2), and canakinumab (n = 2). Of the 8037 patients, 374 (4.7%) were readmitted within 7 days of discharge and received additional anti-inflammatory therapies, presumably because of IVIG-resistance or development or worsening of CAAs.

The number of patients with KD treated at each individual site varied from 30 to 577 (Figure 3, A). The rate of adjunctive therapy with any anti-inflammatory medication was widely variable, with adjunctive agents administered to a range of 14.8%-85.6% of subjects (median, 26.5%) (Figure 3, A and B). The distribution of age groups and male patients was similar across the 45 hospitals (Figure 4). However, there were marked differences in ethnicity and race depending on geographic location.

**Figure 3:**
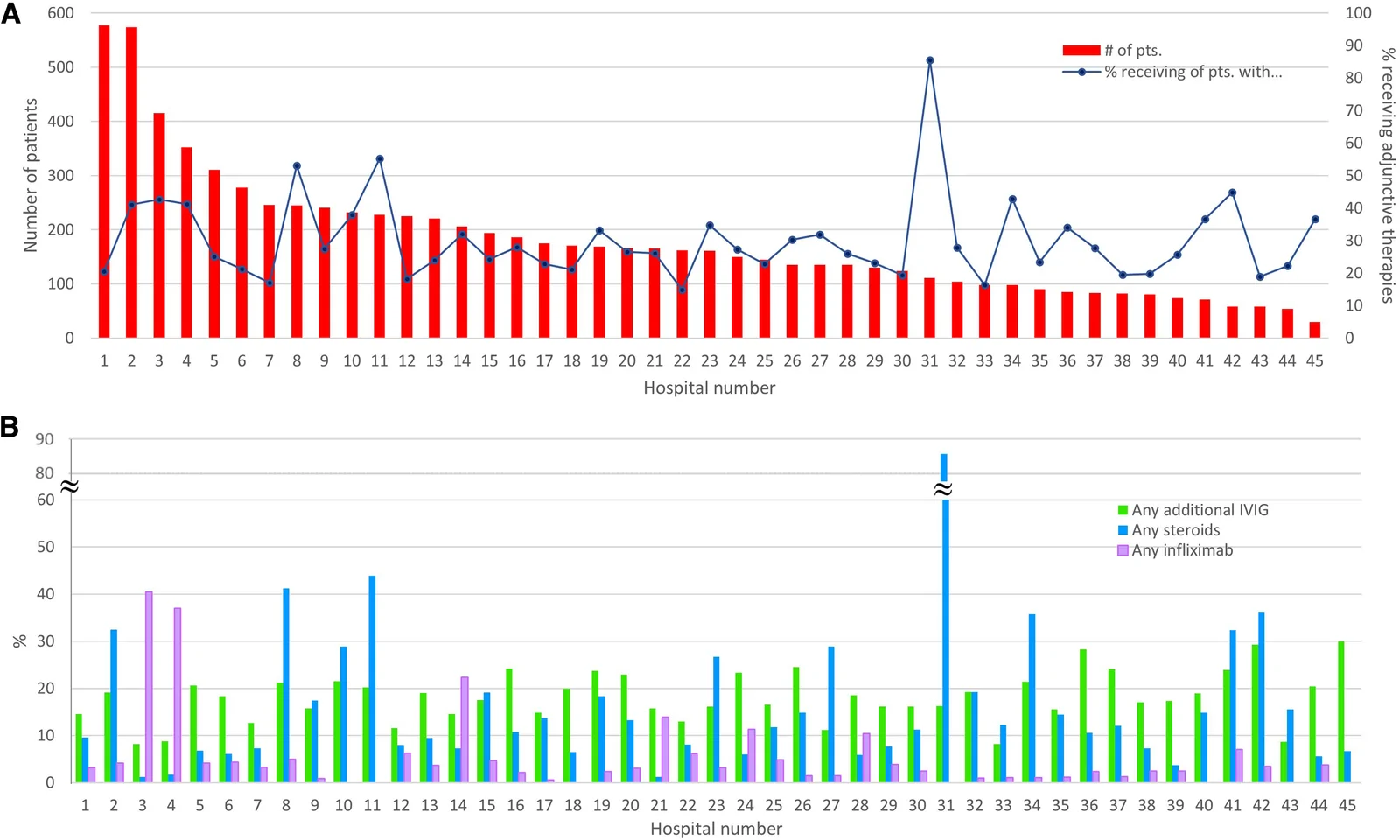
Treatment characteristics by hospital. **A,** Number of patients and frequency of adjunctive therapies. **B,** Percentage of patients for type of adjunctive therapies.

**Figure 4:**
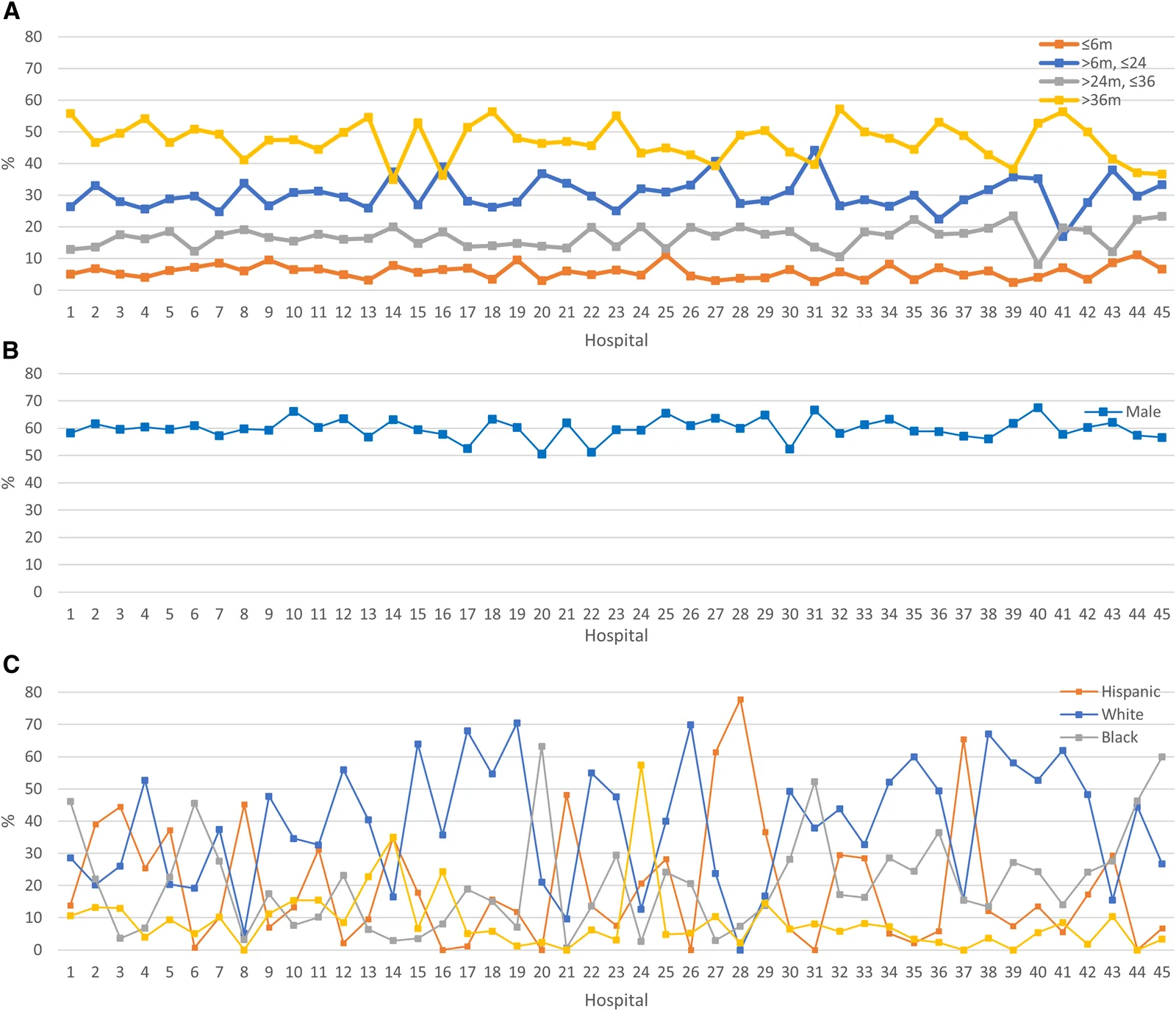
Demographic information of patients in 45 hospitals. **A,** Percentage of age groups (<6 months, ≥6 months and <1 year, 1-4 years, and ≥5 years), **B,** Percentage of males, **C,** Percentage of ethnic/race (Hispanic, non-Hispanic White, Black, and Asian).

To understand the variation in the use of adjunctive treatment by geographic region and year of treatment, the top 4 combinations of additional treatment (additional IVIG, corticosteroids, additional IVIG and corticosteroids, and IFX) were plotted on the US map by year (Supplemental Figure; available at www.jpeds.com). There were marked differences by geographic region, with a higher frequency of corticosteroid use in the Eastern region and IFX use in the Western region. There was a gradual increase in IFX use in 2018 and 2019 in the West, East, and South Atlantic regions.

### Treatment of Patients with CAAs with Anticoagulation

Of the 8037 patients with KD, 822 (10.2%) were coded as having CAAs. Of these, 138 patients (16.8%) were treated with systemic anticoagulants after IVIG treatment and evaluated as suspected patients with gCAAs (Table II). No patient received direct oral anticoagulants during hospitalization in this study period. Of the 138 patients with CAAs and anticoagulation who were presumed to have gCAAs, 38 (27%) were <6 months old and had the highest rate of this complication (Table II, *P* < .0001). Hispanic patients were overrepresented among patients with gCAAs with anticoagulation (32% of patients compared with 22% of other patients with KD, *P* = .01), while Black patients were underrepresented (8% of patients compared with 20% of other patients with KD, *P* = .0003). Males were also overrepresented in the gCAAs with anticoagulation group (71% vs 60%, *P* = .008).

**Table II:** Demographic and clinical characteristics of 138 patients with suspected gCAAs (CAA ICD code associated with anticoagulation treatment)

Characteristics	gCAAs	Other KD	*P* value
	n = 138	n = 7899	
Male, n (%)	98 (71)	4716 (60)	.008
Median age in years (IQR)	1.3 (0.4-3.6)	2.9 (1.5-5.0)	<.0001
Age group, n (%)			
≤6 mo	38 (27)	431 (6)	<.0001
>6 mo and ≤24 mo	45 (33)	2360 (30)	
>24 mo and ≤36 mo	15 (11)	1287 (16)	
>36 mo	40 (29)	3821 (48)	
Race and ethnicity, n (%)			.0006∗
White, non-Hispanic	51 (39)	2825 (37)	
Hispanic	42 (32)	1706 (22)	
Black	11 (8)	1569 (20)	
Asian	14 (11)	796 (11)	
Other or mix	22 (11)	743 (10)	
Unknown	6	260	
Year of onset, n (%)			
2015	29 (21)	1560 (20)	NS
2016	28 (20)	1557 (20)	
2017	30 (22)	1621 (21)	
2018	32 (23)	1612 (20)	
2019	19 (14)	1549 (19)	
Census region, n (%)			
West	46 (33)	2174 (28)	NS
Midwest	26 (19)	1686 (21)	
South	50 (36)	3012 (38)	
Northeast	16 (12)	1027 (13)	
Days of hospitalization, median (IQR)	8 (5-13)	4 (3-5)	<.0001
Number of echocardiograms during hospitalization, n (%)†			
1	19 (14)	5242 (71)	<.0001
2	36 (27)	1581 (21)	
≥3	80 (60)	582 (8)	
Missing info	3	494	
ICU, n (%)	34 (25)	681 (9)	<.0001
Mechanical ventilation, n (%)	9 (7)	101 (1)	<.0001

∗ *P* value calculated for all groups using χ² test. Hispanic vs others: *P* = .01, Black vs others: *P* = .0003. *P* values calculated using a χ² test for categorical values and *t* test for continuous variables.

† Two hospitals were excluded due to missing >50% of echocardiography data; percentage was calculated using patients with KD with echocardiography information.

We analyzed the use of adjunctive anti-inflammatory therapies in this subset of 138 patients with gCAAs. The most commonly used therapies in this group were second IVIG infusion, corticosteroids, and IFX (Figure 5, A). During their initial hospitalization, 42 patients (30.4%) with presumed gCAAs received no additional anti-inflammatory therapies (Figure 5, B). Thirty-seven patients (26.8%) with gCAAs were treated with initial IVIG and anticoagulants on the same day, which suggested that gCAAs were likely present at the time of diagnosis (Figure 5, C). More than half of the patients with gCAAs were recognized and treated with anticoagulants within the first 3 days after initial IVIG administration (Figure 5, D).

**Figure 5:**
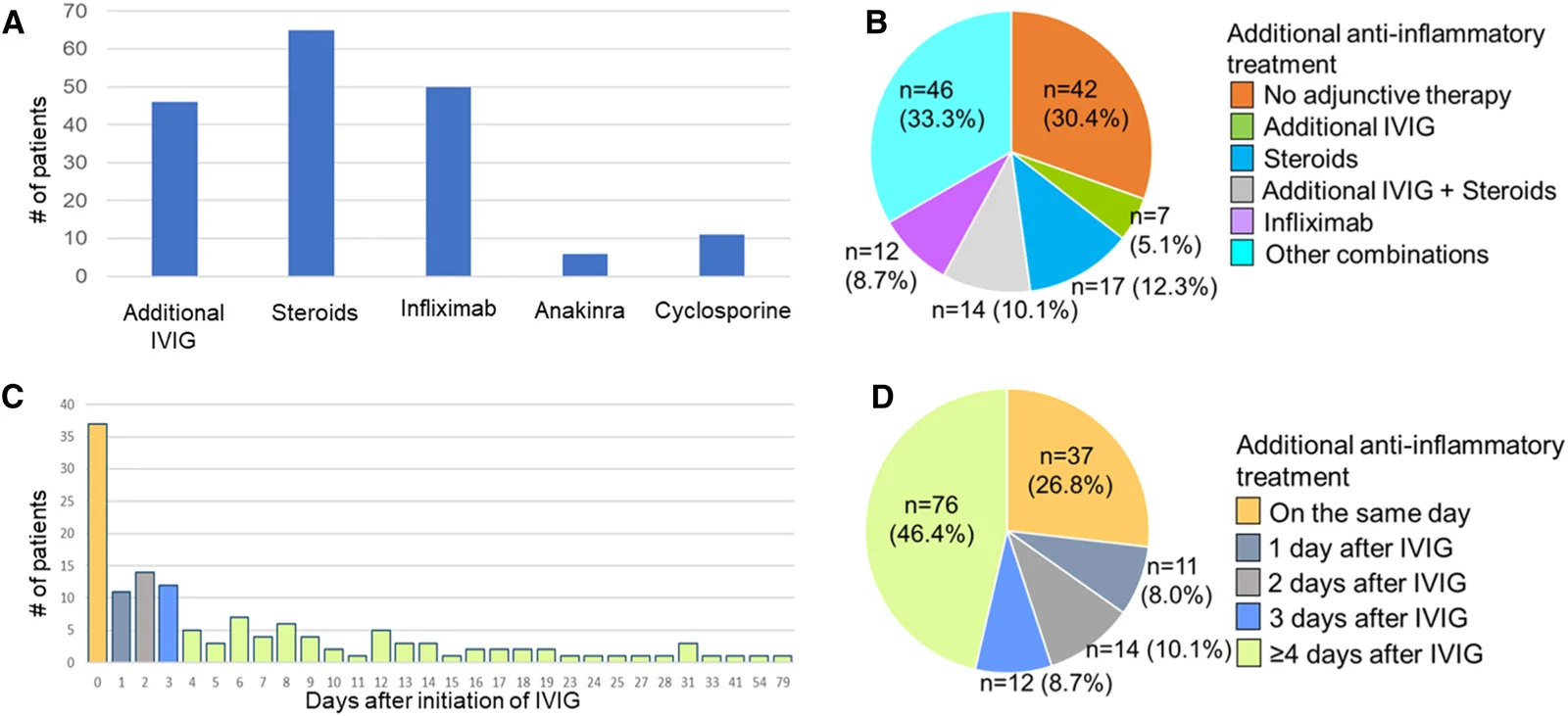
Additional anti-inflammatory treatment of patients with gCAAs. **A,** Number of patients treated with each adjunctive therapy. **B,** Additional anti-inflammatory treatment. **C,** Number of patients with gCAAs by days anticoagulants given after IVIG. **D,** Pie chart of data in **C**.

### Validation of PHIS Data

Of the 415 patients treated for acute KD at our institution during the study period, we identified 412 patients (99.2%) in the PHIS database. Conversely, 412 of 418 (98.5%) patients identified by PHIS were confirmed patients with KD in our local database. The 6 incorrectly identified patients from PHIS were patients for whom the diagnosis of KD had been considered and IVIG given but who were subsequently diagnosed with other conditions. The ultimate diagnoses in this subset of patients were TNF Receptor-associated periodic fever syndrome, polyarteritis nodosa, Takayasu arteritis, megakaryoblastic leukemia, acute lymphocytic leukemia, and 1 patient with fever and an anatomic variant of coronary artery that was initially thought to represent an aneurysm.

Next, we investigated whether we could capture patients with CAAs using the CAA code alone. Of 85 patients with CAAs (RCA or LAD *z* score ≥2.5) at our institution, only 46 patients (54%) had the CAA code (ICD-10 I25.41, R93.1, and ICD-9 414.11) recorded in the PHIS database. Evaluation of patients coded as gCAAs by PHIS using anticoagulant therapy as a proxy for gCAAs revealed that all 3 subjects identified via PHIS were confirmed patients with gCAAs in our local database. One patient with gCAAs in our local KD database was not captured in the PHIS database. This patient had worsening aneurysms, despite adjunctive therapy with IFX and cyclosporine after IVIG, and was subsequently started on apixaban as an outpatient. This patient's outpatient apixaban was not captured by the PHIS database, which only records inpatient data.

## Discussion

Over a 5-year period, 8037 patients with KD were diagnosed and treated at the 45 hospitals that belong to the PHIS. Our analysis provides a snapshot of the diagnosis and treatment at leading pediatric medical centers across the US and reveals different patterns of seasonality by age and region and wide variations in the use of adjunctive treatments among these institutions. For patients with KD having gCAAs, this study highlights the variable use of additional anti-inflammatory therapies, which is a focus of the recent, updated AHA statement.^8^

Our study revealed significant variation in the seasonal cycle by age and location. This intriguing geospatial and temporal effect supports the hypothesis that different triggers for KD are operative during different seasons and at different locations. A large-scale epidemiologic study of patients with KD from the Japanese National Survey revealed distinct age-specific patterns of KD incidence.^4^ In Japan, infants aged <6 months had a distinctly different epidemiology from older children and adolescents. Toddlers (ages 6-<24 months) exhibited a large change in their seasonal cycle in the mid-2010s, and children with ages ≥36 months had similar patterns of seasonality. Those data suggested potential social or behavioral factors associated with exposure changes. Therefore, we applied the same age classification in this study. Interestingly, the lack of clear seasonality in infants <6 months was observed in both the Japanese and US cohorts, though the specific patterns varied slightly. Although the Japanese cohort exhibited a more distinct summer peak, the PHIS cohort showed a more erratic distribution of cases throughout the year. These discrepancies may result from differences in sample size and study duration, or they may suggest that the environmental triggers for KD in infants vary by geographic region and population. Although a relative peak was observed in August and September in our current study, this reflects a decrease in cases among older children during summer months rather than an increase in infant cases. These studies suggest that KD triggers may differ for infants <6 months and older children for whom social practices such as daycare and school attendance may influence the exposure to different KD triggers. Infants may have either a different mode of exposure to the causative agent or a different trigger likely encountered in the home.

Frequently used adjunctive therapies included a second infusion of IVIG, corticosteroids, and IFX. Addition of prednisolone to IVIG improved coronary artery outcomes in patients with severe KD in Japan (Randomized Controlled Trial to Assess Immunoglobulin Plus Steroid Efficacy for Kawasaki Disease [RAISE] study).^10^ A clinical trial comparing IFX with a second infusion of IVIG for the treatment of resistant KD showed that IFX was safe and more effective than a second IVIG in abrogating fever, shortening hospital stay, and reducing measures of systemic inflammation.^9^ A retrospective, single-center study also showed that the combination of IVIG and 10 mg/kg of IFX led to more rapid resolution of CAAs as compared to IVIG alone or in combination with 5 mg/kg of IFX.^11^ Following the 2017 AHA statement that included IFX as an option for adjunctive therapy, its use increased in the eastern US where corticosteroids were used more frequently. The current study showed that corticosteroids are still the leading adjunctive therapy used for patients with KD. Whether the variation in the use of adjunctive therapy was consistent with the current AHA guidelines was difficult to assess, given that the reasons for adjunctive therapy administration were not available. Approximately 30% of patients with gCAAs did not receive anti-inflammatory therapy beyond their initial IVIG. This percentage should go down because the recent 2024 AHA statement recommends the use of adjunctive anti-inflammatory therapies for patients with an initial abnormal coronary artery *z* score who are at high risk for gCAA development.

To study the characteristics of patients with KD having CAAs, Lo et al used antithrombotic therapies (warfarin, enoxaparin, tissue plasminogen activator, clopidogrel, dipyridamole, and abciximab), while Son et al used CAA coding.^3^^,^^12^ Our local database evaluation revealed that clopidogrel was not reliable for identifying patients with giant aneurysms as clopidogrel is occasionally used to replace aspirin for patients during influenza season to avoid Reye syndrome and also used in dual antiplatelet therapy for moderate-sized aneurysms. We also found that CAA coding alone was unreliable for identifying patients with CAAs as only 54% of our patients with a *z* score ≥2.5 had the CAA code recorded in the PHIS database. Also, CAA code “abnormal findings on diagnostic imaging of heart and coronary circulation (ICD R931)” may catch small to medium aneurysms but may also be used for patients with mitral regurgitation or aortic dilation and not coronary artery abnormalities (CAAs). Therefore, we used both CAA coding and the use of warfarin and/or enoxaparin after IVIG treatment to identify patients with gCAAs. In the comparison of PHIS data with our single-center database, we confirmed all 3 patients identified by PHIS, although 1 patient who progressed to gCAAs as an outpatient was not captured in the PHIS database.

Our analysis revealed variations in the prevalence of gCAAs across racial and ethnic groups, most notably in Hispanic and Black patients. While these findings are noteworthy, the ability to investigate these findings is limited by the granularity of the PHIS dataset. Further study is needed to investigate the patient, provider, and systemic factors that may influence KD treatment patterns and outcomes.

### Limitations

Previous studies have examined KD treatment and seasonality using the PHIS database^1^, ^2^, ^3^ and all the limitations of using an administrative database apply. However, this is the first study to evaluate the accuracy of PHIS data by comparing it to a single-center, carefully curated REDCap database. This comparison showed very high concordance, confirming that the PHIS data accurately capture patients with acute KD. However, it is likely that coding omissions and other errors still exist in the PHIS database.

The Centers for Disease Control and Prevention reported that in 2019 more than 5000 children under 18 years of age were hospitalized with KD in the US (https://www.cdc.gov/kawasaki/about/index.html). Although we cannot know how many of these patients were acute cases treated with IVIG, this number is more than 3 times greater than the number reported by the PHIS (n = 1568 in 2019). This suggests that the majority of patients diagnosed with KD were treated at hospitals not affiliated with the PHIS. Therefore, the snapshot of KD care presented here presents only a subset of patients with KD across the US. Also, the data collected from the PHIS are only from inpatients. Because 3 hospitals contributed less than 5 years of data and there were several trials of adjunctive therapy in the US during the study period, the temporal and regional variations in secondary therapy should be interpreted with caution. If a patient developed CAAs after discharge or experienced worsening of CAAs after discharge and was treated as an outpatient, we would have missed that patient. Therefore, the CAA and gCAA rates are likely underestimated.

Our determination of acute KD cases used a combination of ICD codes and IVIG treatment to define acute KD. Therefore, patients with recurrent KD were not captured in this study.

The reason for additional anti-inflammatory therapies is not specified in the PHIS database. The illness day of admission and treatment are not captured in the PHIS database so we were unable to evaluate whether patients were treated within the first 10 days after fever onset as recommended by current guidelines. Our sensitivity analysis using single-center data demonstrated that the true incidence of CAAs and gCAAs is likely higher than what was captured in our analysis using PHIS database.

In conclusion, in this national cohort of 8037 patients with KD treated at 45 children's hospitals across the US, we identified significant geographic and age-related differences in seasonal incidence and marked variation in the use of adjunctive therapies in high-risk patients. These findings underscore a critical need for prospective trials to guide standardized treatment approaches.

## CRediT authorship contribution statement

**Chisato Shimizu:** Writing – review & editing, Writing – original draft, Formal analysis, Data curation, Conceptualization. **Laurel DeHaan:** Writing – review & editing, Formal analysis, Data curation. **Shoko Yamazaki:** Writing – review & editing, Data curation. **Kyohka Sato:** Writing – review & editing, Data curation. **Daniel R. Cayan:** Writing – review & editing, Formal analysis. **Jennifer Kim:** Writing – review & editing, Data curation. **Emelia V. Bainto:** Writing – review & editing, Data curation. **Kirsten B. Dummer:** Writing – review & editing, Data curation. **Adriana H. Tremoulet:** Writing – review & editing, Conceptualization. **Jane C. Burns:** Writing – review & editing, Conceptualization. **Michael A. Gardiner:** Writing – review & editing, Conceptualization.

## Declaration of Competing Interest

This work was supported by “The Gordon and Marilyn Macklin Foundation”.

The authors declare that they have no known competing financial interests or personal relationships that could have appeared to influence the work reported in this paper.
