# Supplementary material for: Variability in Diagnosis and Treatment of Kawasaki Disease across the United States
Source: J Pediatr Clin Pract. 2026 Aug 17;22:200237. doi: 10.1016/j.jpedcp.2026.200237 (PMC13629332; doi:10.1016/j.jpedcp.2026.200237)
Supplement: Supplementary file 1 [file mmc1.docx]

**Supplemental Figure. Adjunctive therapies by census division and year**
